# Biomechanical Properties of Bone and Mucosa for Design and Application of Dental Implants

**DOI:** 10.3390/ma14112845

**Published:** 2021-05-26

**Authors:** Michael Gasik, France Lambert, Miljana Bacevic

**Affiliations:** 1 School of Chemical Engineering, Aalto University Foundation, 02150 Espoo, Finland; 2 Dental Biomaterials Research Unit, University of Liege, 4000 Liège, Belgium; france.lambert@chu.ulg.ac.be (F.L.); miljanabacevic@gmail.com (M.B.)

**Keywords:** bone, soft tissues, dental implants, biomechanics, in silico, stiffness

## Abstract

Dental implants’ success comprises their proper stability and adherence to different oral tissues (integration). The implant is exposed to different mechanical stresses from swallowing, mastication and parafunctions for a normal tooth, leading to the simultaneous mechanical movement and deformation of the whole structure. The knowledge of the mechanical properties of the bone and gingival tissues in normal and pathological conditions is very important for the successful conception of dental implants and for clinical practice to access and prevent potential failures and complications originating from incorrect mechanical factors’ combinations. The challenge is that many reported biomechanical properties of these tissues are substantially scattered. This study carries out a critical analysis of known data on mechanical properties of bone and oral soft tissues, suggests more convenient computation methods incorporating invariant parameters and non-linearity with tissues anisotropy, and applies a consistent use of these properties for in silico design and the application of dental implants. Results show the advantages of this approach in analysis and visualization of stress and strain components with potential translation to dental implantology.

## 1. Introduction

The placement of a dental implant system for the restoration of a missing tooth or teeth relies on the osseointegration of the implant (screw and abutment) with the maxillary or mandibular bone, whereas abutment part success depends on its adherence and integration with soft mucosal (gingival) tissue [1,2,3]. The dental implant is exposed to different mechanical stresses from swallowing mastication and parafunctions (grinding, clenching) via the application of the forces to the crown for a normal tooth, leading to simultaneous mechanical movement and deformation of the whole structure. Depending on the degree of the osseo- and mucosal integration achieved to the time point, this mechanical force might be or might not be fully transferred to the bone and mucosa.

Non-optimal displacements can have an undesirable effect on the integrity of biomaterial-tissue interface, which serves as a barrier that prevents bacterial penetration [4,5,6], and a subsequent peri-implant disease or even implant loss. Therefore, the formation of an early and long-standing biomaterial tissue interface is of paramount importance. Natural tooth and the implant regulate and stimulate respective, although not identical, biological mediator to heal by promoting the tissue remodeling process [7,8].

Hence, the knowledge of mechanical properties of the bone and gingival tissue in normal and pathological conditions is very important for the successful conception of dental implants to access and prevent potential failures and complications originating from incorrect mechanical factors combinations.

Numerous studies were carried out and clinical data have been collected regarding the shape, design, surface and geometry of abutments and dental screws and their suitability for different tissues’ quality and location [1,2,3,9,10,11,12,13]. Significant differences were also reported for seemingly identical implanted materials but originated from different sources [14]. Suitable for clinical use, the mechanical characterization of bone and soft tissues is more problematic than for metallic, ceramic and polymer materials. Many datasets published are lacking consistency and details about the measurement protocols and conditions. The generalization of these data is very difficult or almost impossible when it comes to providing simple, robust and relevant information which can be integrated with modern 3D/4D imaging and the associated planning of operations.

Basically, there are two extremes in analyzing and documenting the mechanical properties of materials and tissues. The simplest one treats all materials as linear elastic or viscoelastic matter to approximate the properties into single numbers usually referred to as “elastic modulus”. The elastic modulus definition, however, fits only linear elastic materials for very small deformations, as was set by the theory of elasticity for centuries. The guidelines of the National Physical Laboratory (UK) list nine methods of calculation of elastic modulus [15], all of them leading to different values. 

Almost all biomaterials and tissues are clearly not elastic ones, so it is a significant oversimplification to try to artificially reduce data to some fixed numbers [16]. The advantage of the linear elastic models is in provision of simple direct prediction of the tissues’ properties for the sake of computational efficiency. This is why such models have been extensively adopted in a range of studies and in the clinical field. One however might question the usefulness of these data: what is the benefit of knowing “elastic modulus of mucosa” ranging 0.1~680 MPa [17] or “elastic modulus” of periodontal ligament (PDL) spanning 0.07~1750 MPa [18,19]?

Another option takes a very sophisticated approach, such as hyperelastic models or phenomenological quasilinear viscoelasticity [20,21], with a substantial number of artificial fitting parameters. For example, Ogden’s energy function for PDL may require three different shear moduli, three non-linearity exponents and three initial bulk moduli, being calculated for three principal axes [22]—even 15 fitting parameters for anisotropic elastic potential are required, even when using invariants [23,24]. There are great experimental difficulties to make such tests within existing standards, protocols, and ad hoc test methods [20]. Review [25] lists about 150 methods of testing of thermal cycling for dental restorations, with a great incompatibility between different data.

Linearization of the hyper-elastic models can generate many rather different “elastic moduli”, and in experiments aiming for clinical relevance, the curve taken might be far away from the conditions of physiological activity [26]. It was emphasized [19] that any constitutive equation of the tissue behavior ideally should be sufficiently robust, with parameters reflecting true material properties and not just a “best fit” function.

Sophisticated multi-parameter fitted models [20,22,23] have empirical coefficients with a little or nonphysical meaning. This contrasts constitutive equations, where parameters were derived from physical principles with the coefficients being causal, determinable and quantifiable material functions [19,20,27]. Such equations are, in general, influential in predicting the stresses and strains developing in the oral cavity linked to the forces and displacements, due to normal physiological conditions, indicating possible risks of tissue failure [19].

The focus of this study is to analyze known experimental and modelled data for bone and oral soft tissues, to present more convenient computation methods, incorporating tissue anisotropy, and to present a consistent use of these biomechanical properties for in silico design and the analysis of dental implant application.

## 2. Literature Data Assessment

### 2.1. Reported Bone Properties

Both cortical and trabecular (cancellous) bone properties have been extensively analyzed in vivo and ex vivo, with a variety of static and dynamic methods [11,12,13,20,21,22,28,29,30,31]. Some studies were focused more on simplified engineering values, whereas others also considered bone anisotropy and microstructure effects in more sophisticated detail. Several studies also incorporated the effect of bone quality (expressed as density, mineral content, permeability, etc.) on bone mechanical properties. The most-used data of the elastic modulus of bones are shown in Table 1. 

More complex correlations of specific bone stiffness tensor components vs. bone volume fraction, mean intercept length of the microstructure and applied strain rate can be found in literature [21], but such detailed analysis is usually local and not possible to be made in vivo. Here, a primary look is on such data on bone anisotropy, which could be deployed in clinical practice with reasonable tolerances [31].

Variations and differences between the data obtained with different test methods for bone and other tissues are well known. This makes it difficult to decide the values to be used for the design and placement of the dental implants. From a biomechanology point of view [20], the most relevant data are those obtained in conditions corresponding closely to the host physiological ranges. 

The values obtained in more exotic conditions (high frequency ultrasound, nanoindentation, etc.) are correct for those selected methods and environments, but their practical use is inferior to numbers measured at the situations serving clinical application purposes (e.g., at 1 Hz with 30–100 μm amplitude [22]). Carrying out such “right” tests, however, is not always easy. Even in simple setups like uniaxial compression or simple bending, orientation anisotropy of the bone specimen might be difficult to account properly.

### 2.2. Reported Oral Soft Tissues Properties

The experimental testing of viscoelastic properties of dog, porcine and human mucosa and other oral section (palate, gingiva, periodontal ligaments) *in vivo* and *in vitro* was carried out or summarized in many studies [17,19,26,34,35,36,37,38,39,40]. As expected, there is a significant scatter in the data (Table 2)—more than for bones—depending on tissue location, conditions, and the deployed testing method.

Many studies do not provide a well-defined rationale as to why these specific parameters have been chosen for the tests and how other parameters’ selection might have affected the data obtained. Furthermore, as stated in [21], the testing of isolated soft tissues presents substantial challenges, due to preparation variations, grips and clamping effect (especially in tensile and shear modes), the inaccurate measurements of geometry and cross-sectional areas (for stresses estimation), preconditioning methods, deformation rates, etc.

In rheology (such as oscillatory shear), even more errors and artefacts are often seen in the literature data. These may comprise, i.a., *a priori* set linear viscoelastic (LVE) models (not suitable for biological tissues), wrong boundary conditions in real tests vs. models; ignorance of present momentum diffusion or viscoelastic waves; disturbances from secondary flows, especially at low Reynolds numbers (considered as “laminar flow”); non-controlled elastic instabilities at high Weissenberg numbers; waves propagation in dynamic tests [9,20,27]. This makes the selection and comparison of consistent data values for soft tissues much more difficult than for a bone.

It is known that soft tissues including oral ones (mucosa, gingiva, palate, PDL) exhibit complex nonlinear time-dependent behavior [17,39,41,42]. However, most of the models in literature assume material and geometric linearity with the homogeneity and isotropy of the mucosa. Due to evident limitations of such approach, more complex models such as hyperelastic ones were deployed in description of these soft tissues (a hyperelastic material modelling formulates a potential energy function (strain energy potential) per unit of reference volume [17] as a function of the strain at a typical point in the material). This function can be dependent either on strain tensors of a nonlinear deformation field, on the invariants of these strain tensors, or directly on the principal stretches [17].

However, hyperelastic material model application is limited, especially in its demand for a substantial number of parameters or fitting functions if nonlinearity and anisotropy have to be included in the calculations [22,23,43]. It is difficult to estimate to what extent these fitting values could be translated to other subjects with different heterogeneous anatomical microstructures or generalized for practical use under complex physiological responses.

### 2.3. Other Parameters of Clinical Importance

Among the parameters which might be treated as having significant clinical relevance, osseointegration quality improvement and the minimization of the pressure–pain threshold (PPT), interstitial fluid (hydrostatic) pressure (IFP) and pressure leading to residual ridge resorption (destruction of the supporting bony tissues) can be mentioned.

Osseointegration is a complex outcome (or an endpoint) which can be evaluated only after some time (from months to years), combining objective measures from an examination, as well as patient satisfaction with physiological functionality and aesthetic effect (“quality of life”). Other outcomes can be, in principle, calculated or estimated using experimental and modelling (in silico) data. The biomechanical models of “hard” (bone) and “soft” (mucosa) tissues aim to interpret, analyze and (where possible) predict various aspects of these tissues’ responses to dental prostheses [17].

It is also known that the success of a dental implant depends on a variety of biomechanical factors, including the design and position of the implant, implant-abutment connection, cantilever length, surface roughness, bone quality and type, depth of insertion, arch configuration, the nature of bone–implant interface, and occlusal conditions [1,2,9,12,14,29]. Whereas such biomechanical factors have been simulated earlier, the results are often contradictory, due to differences in model parameters, construction, materials models selection and meshing [29].

In regard to mechanical stimuli, many reported studies are expressing acting stress in an equivalent format such as von Mises stress [17,29,31,33,44]. However, the use of von Mises stress has substantial limitations: it has been most widely applied for engineering problems as a yield criterion, stating that the yielding of a material occurs once the second deviatoric stress invariant reaches a critical (yield stress) value [17,21,31,45].

Yielding (nonelastic irreversible deformation) is clearly defined for metals, plastic and ceramic materials, but it is questionable for live dental tissues. Furthermore, since von Mises stress is a module (absolute value) of the deviatoric stress, it is always positive: one cannot see from the von Mises data whether the area or point in question is under tension or compression.

Because the tissue’s behavior in tension and compression is different and often non-linear, the use of symmetric stress characterization is a clear oversimplification. Indeed, authors [29] noted that besides single values alike von Mises stress, the magnitudes of the stress concentrations, stress distribution, and displacement components of specific points should be analyzed as they could provide a valuable information about the deformation of the model and assist in the interpretation of the results.

For example, micromovements along the bone implant interface must stay within proper tolerances, as micromotions beyond those tolerances result in connective tissue encapsulation or adherence failure [1,2,9,11]. It is thus preferable to see whether there are tensile, compressive and shearing areas to identify possible risks. This could be done by visualizing selected stress and strain tensor components.

The average data for PPT identified by different studies are usually within a 0.1~0.4 MPa range [17]. Most of these data were acquired for the cases of dentures on mucosa, and it is not clear which pressure level inside the bone tissue would be considered as one causing a noticeable pain. It is known than cortical bone can withstand much higher pressures, since bone permeability is low, and respectively its physiological perfusion requires a significant pressure gradient [16,21,30].

For hydrostatic pressure (IFP) values between 0.3 to 2 kPa in gingival mucosa have been reported according to data [17]. Authors [46] have reported a “safe” level of internal pressures for soft tissues <5~10 kPa (preferably <4 kPa) and for a short-term loading <13~20 kPa: once it exceeds the vascular pressure differential, blood flow will be reduced and may temporarily cease, potentially leading to local anoxia and localized ischaemia [16,46,47,48]. Both PPT and IFP can be, in principle, calculated with in silico FEA post-processing and compared with the criteria above to understand what potential risk might be associated in different locations.

For residual ridge resorption, about 20 kPa of intermittent pressure and 7 kPa for continuous pressure at mucosa were reported to cause alveolar bone ridge resorption [17]. These data are compatible with the one shown above for IFP (<20 and <5~10 kPa) respectively [46], so that they might be recommended as watch criteria in results analysis.

The summary of these parameters, which would be qualified as those for “acceptable clinical risks”, could be highlighted as shown in Table 3. These values could be recommended to monitor in simulations and the optimization of the personalized implants design.

## 3. Methods

### 3.1. Bone Stiffness Approximation

Bone and other biological tissues are non-linear viscoelastic materials, so for time-dependent, direction- and strain-rate dependent behavior, rather complex constitutive equations have to be used. Usually, the tissue behavior of an anisotropic viscoelastic material is described based on the application of the Boltzmann superposition integral [21,31], with (time-dependent) second rank stress (*σ_ij_*) and strain (*ε_ij_*) tensors and four-rank stiffness tensor *T_ijkl_.* This links stress (σ) and strain (*ε*) of the material as:

(1) $$\sigma =T\;\times \;\;\varepsilon ,\;S\;\;=T^{-1}$$

where **T** is the stiffness tensor and **S** is the compliance tensor (the inverse of T). For the reduced notation of **T** leading to the 2-rank stiffness tensor **C**, there are 36 remaining independent elements (2) for the lowest symmetry case and 12 nonzero independent elements (*C*_11_…*C*_33_, *C*_44_, *C*_55_ and *C*_66_) for an orthotropic solid [21,31,49].

(2) $$\left\|C\right\|=\left(\begin{array}{cccccc} C_{11} & C_{12} & C_{13} & C_{14} & C_{15} & C_{16}\\ C_{21} & C_{22} & C_{23} & C_{24} & C_{25} & C_{26}\\ C_{31} & C_{32} & C_{33} & C_{34} & C_{35} & C_{36}\\ C_{41} & C_{42} & C_{43} & C_{44} & C_{45} & C_{46}\\ C_{51} & C_{52} & C_{53} & C_{54} & C_{55} & C_{56}\\ C_{61} & C_{62} & C_{63}^{} & C_{64} & C_{65} & C_{66} \end{array}\right)\;;\;\sigma =C\;\;\times \;\;\varepsilon$$

Resulting stress-strain equations for anisotropic materials are easily becoming rather cumbersome, requiring a special computation to be used in the practice. Nevertheless, for the more precise planning of the implant and its expected mechanical stability it is important to know the anisotropy of the combined cortical and trabecular (cancellous) bone as a whole, since the screw displacements and deformations have to be compliant to both bone tissues at the same time. As the abutment is tightly fixed to the screw, the movement of the screw and the abutment are highly correlated: they are moving together as a single assembly object.

Abutment purpose is to have sufficiently tight adhesion to the local gingival tissue laying on the bone, so both gingiva and the bone would face the same resulting displacement of the fixture, but this displacement will be felt differently by bone and gingiva, due to intrinsic differences in their properties. Hence, the same displacement will result in different strains in gingiva, cancellous and cortical bones. Due to properties’ anisotropy and non-linearity, it is improper to describe all these players with single values of “elastic modulus”.

Most of the finite element analysis (FEA) software uses an assumption that the bone is linearly elastic isotropic material, which cannot catch the differences in local strains and stresses faced by the tissues [33]. For anisotropic materials instead of “elastic modulus”, the whole stiffness matrix (2) needs to be considered. The latter can be simplified for higher symmetry materials like an orthotropic one, when many off-diagonal components (3a) vanish [45,50]. There compliance matrix **S** is shown in (3a) is in the reduced notation for orthotropic symmetry, with *E_ii_* being directional elastic moduli (11 = X, 22 = Y, 33 = Z directions as in Table 1) and *G_ij_* are respective shear moduli [45]. The Poisson ratios (*ν*) in general are not symmetric *ν_ij_* ≠ *ν_ji_* (3b).

(3a) $$s=\left\|\begin{array}{cccccc} 1/E_{11} & -\nu_{12}/E_{22} & -\nu_{13}/E_{33} & 0 & 0 & 0\\ -\nu_{21}/E_{11} & 1/E_{22} & -\nu_{23}/E_{33} & 0 & 0 & 0\\ -\nu_{31}/E_{11} & -\nu_{32}/E_{22} & 1/E_{33} & 0 & 0 & 0\\ 0 & 0 & 0 & 1/G_{23} & 0 & 0\\ 0 & 0 & 0 & 0 & 1/G_{31} & 0\\ 0 & 0 & 0 & 0 & 0 & 1/G_{12} \end{array}\right\|$$

(3b) $$\nu_{ij}=\{\begin{array}{c} -S_{ij}/S_{ii}\;if\;i\neq j\\ 1\;\;\;if\;i=j \end{array}$$

In Table 1, reported values of the stiffness matrix components are those usually deployed in bone behavior modeling. The coordinate indexing adopted here is as commonly used, i.e., it takes X- (11), Y- (22) and Z- (33) orthogonal directions [45,49] and cross-components as XY (12), YX (21), YZ (23), etc., with a row normalization (3a, 3b). The allocation of global coordinates is usually as Z for infero-superior direction, Y for bucco-lingual direction, and X for mesio-distal direction [33]. Local coordinates, which describe the local instant direction, depend on the point of view (=they can be rotated), keeping the orthogonality and alignment with the tissue structure.

**Figure 1:**
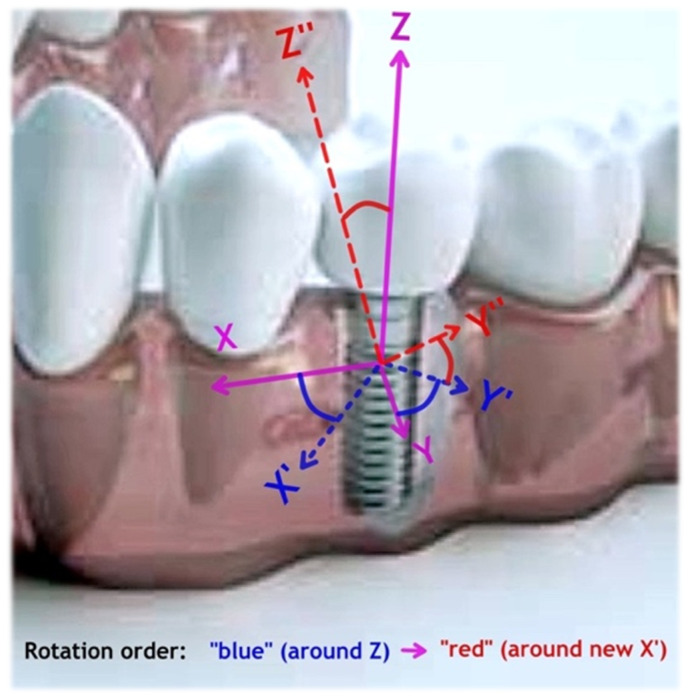
Schematic of the implant in the mandible and respective coordinate axes with rotations as shown in the text. The first rotation (“blue” with the yaw angle Ψ) around Z-axes is followed by the second (“red” with the pitch angle Θ) around new X’-axes counter-clockwise.

The rotation of the coordinates for expression of the 4-rank tensor components *T_ijkl_* can be performed with two consequent rotations [50], as shown in Figure 1. There original position of Z-coordinate is aligned with the axis of the implant in the superior direction, Y-coordinate in the buccal direction, and X-coordinate in distal direction. The first rotation (shown in Figure 1 as blue axis) is done around Z-axes by yaw angle Ψ, resulting in new X’ and Y’ axes. The second rotation (shown in Figure 1 as red axis) is done along this new X’ axis by pitch angle Θ. This leads to a new set of local coordinate axes X’–Y”–Z”, which can be directed to any point of view just by these two rotation operations (note that the order of rotation is important [50], Figure 1).

Finally, to calculate new rotated stiffness components, the unrotated ones are to be multiplied with the rotation matrix **Q** (4b) and summed over as in (4a). The rotated stiffness tensor and its new inverse (compliance tensor) can now be used for the calculation of anisotropic stresses and strains in the implant-bone-gingiva system.

(4a) $$T_{ijkl}^{rot}=\sum_{p=1}^{3}\sum_{q=1}^{3}\sum_{r=1}^{3}\sum_{s=1}^{3}Q_{pi}Q_{qj}Q_{rk}Q_{sl}T_{ijkl},$$

(4b) $$Q(\Psi ,\Theta )=\left(\begin{array}{ccc} \cos\Psi \cos\Theta & \sin\Psi & -\cos\Psi \sin\Theta \\ -\sin\Psi \cos\Theta & \cos\Psi & \sin\Psi \sin\Theta \\ \sin\Psi & 0 & \cos\Theta \end{array}\right)$$

Before using known data which consist of separate elastic and shear moduli (Table 1 and Table 2), they must be converted first into the stiffness tensor **C** components. Here these data of elastic moduli (*E_i_*, *G_ij_*) were converted with Equation (4) into the reduced stiffness matrix components *C_ij_* and respective Poisson ratios *ν_ij_* using “RotaStiff” software developed by the author [50]. This conversion can also be done even with a spreadsheet, but such computations are more cumbersome.

The rotation (4b) does not lead to a symmetric tensor (*C_ij_* ≠ *C_ji_*) also due to differences in Poisson’s ratios (for example, for data [33] one has *C*_21_ = 8.169 and *C*_12_ = 8.162, and *C*_13_ = 9.678 vs. *C*_31_ = 9.725 GPa, Table 4). For practical applications, specific tissue properties of the patient are desired instead of averaged literature values, despite the latter still being able to be used as a good approximation if their scatter in values is acceptable.

Most of the differences in “bone quality” measured using X-ray or CT examination are related to the bone density [33]. It was shown [33,51,52] that for cancellous bone, its apparent density *ρ_a_* in g/cm^3^ (without bone marrow, which does not contribute to load bearing capacity) can be rather well calculated from CT contrast values in *HU* (Hounsfield units) as:

(5) $$\rho_{a}=1.011\cdot 10^{-3}\;\times HU$$

Together with the experimental values reported in [53] and using the conversion of single elastic properties into the stiffness matrix, authors have obtained the following stiffness matrix (GPa) for cancellous mandibular bone as functions of *ρ_a_*:

(6) $$C_{canc.bone}=\left(\begin{array}{cccccc} 2.623\rho_{a}^{2.15} & 0.191\rho_{a}^{2.15} & 0.847\rho_{a}^{2.15} & 0 & 0 & 0\\ 0.019\rho_{a}^{2.12} & 1.276\rho_{a}^{2.12} & 0.019\rho_{a}^{2.12} & 0 & 0 & 0\\ 0.07\rho_{a}^{} & 0.0157\rho_{a}^{} & 0.2167\rho_{a}^{} & 0 & 0 & 0\\ 0 & 0 & 0 & 0.631\rho_{a}^{2.12} & 0 & 0\\ 0 & 0 & 0 & 0 & 0.073\rho_{a}^{} & 0\\ 0 & 0 & 0 & 0 & 0 & 1.113\rho_{a}^{2.15} \end{array}\right)$$

This allows a direct computation of the stiffness matrix from the *HU* values (5). For cortical bone, a similar approach can be used, and with data [44,54], authors have obtained the following stiffness matrix values for mandibular cortical bone (GPa) vs. bone true (not apparent) density *ρ* (g/cm^3^):

(7) $$C_{cort.bone}=\left(\begin{array}{cccccc} 8.35\rho +0.39 & 3.62\rho +0.17 & 2.87\rho +0.134 & 0 & 0 & 0\\ 7.83\rho -7.84 & 17.73\rho -17.80 & 6.135\rho -6.154 & 0 & 0 & 0\\ 18.47\rho -18.32 & 18.32\rho -18.23 & 32.83\rho -32.70 & 0 & 0 & 0\\ 0 & 0 & 0 & 2.44\rho +0.11 & 0 & 0\\ 0 & 0 & 0 & 0 & 5.18\rho -5.20 & 0\\ 0 & 0 & 0 & 0 & 0 & 9.26\rho -9.23 \end{array}\right)$$

For both cancellous (6) and cortical (7) bones stiffness, components *C_ij_* are not symmetrical, and they depend on bone densities in different ways.

### 3.2. Soft Tissues Stiffness Approximation

As follows from Table 2, there is a great scatter in the soft tissue data depending on the origin, preparation, testing method and the interpretation of the data. There is also a natural difference in tension, shear and compression method outcomes and it is not very clear which of these data are actually relevant for in vivo situation. When the implant is loaded in the physiological range, the displacements are usually below 50–100 μm along the direction of applied force [22]—micromovements over 150 μm are to be avoided [55]. During these micromotions, gingival tissue around the abutment undergoes some mixed deformation mode with local tension, shear and compression at the same time. Based on reported data (Table 2) it is not however possible to artificially derive some averaged properties by combination of tensile and compressive results, which could be directly applicable to this case. Furthermore, there are no reliable data showing the anisotropy of such soft tissues, compared to the data available for the bone (Table 1 and Table 3).

In this analysis, authors have selected the publications that have numerical and graphical data of the stress–strain–time relation for at least one reported experiment with sufficient details (test method, frequency, time, preconditioning, deviations, etc.). These data were digitized with GraphClick software (Arizona Software Ltd., Neuchâtel, Switzerland) and evaluated with BEST software (Seqvera Ltd., Helsinki, Finland) to extract invariant values [24,27]. Some sources in Table 2 were rejected, where only a value or a range was shown without the explanation of the method of analysis or calculation, or which otherwise were lacking essential information. Results where ‘elastic moduli’ or similar property was evidently obtained with the differentiation of stress by strain and extrapolation to zero strain or strain rate, were not analyzed due to larger errors and lesser relevance for clinical applications.

The invariant values [24] were obtained with BEST method [27] and can be used for the estimation and prediction of biomaterials and tissue properties without the use of a material model, as was demonstrated in [9,10,20,56,57]. There, the dependence of the material strain *ε* from the applied stress *σ* under pseudodifferential time-convolution (accounting for loading history in this case):

(8) $$\varepsilon (t,\sigma )=\frac{1}{\Gamma (\alpha )}\overset{t}{\underset{0}{\int }}\;\frac{S\times \sigma (t,\tau )d\tau }{{\left(t-\tau \right)}^{1-\alpha }}=\frac{1}{\Gamma (\alpha )\langle E_{}^{0}{\left(\tau_{}^{0}\right)}^{\alpha }\rangle }\overset{t}{\underset{0}{\int }}\;\frac{\sigma (t,\tau )d\tau }{{\left(t-\tau \right)}^{1-\alpha }}\;$$

where *α* is a material memory value, *E*^0^ is the averaged time-invariant (i.e., not time-dependent) intrinsic modulus, *τ*^0^ is invariant characteristic time, and Γ() is the gamma-function. The product <*E*^0^(*τ*^0^)^α^> is the time-averaged viscostiffness (a pseudo-property) of a material [20,27,46,56,57]. Characteristic invariant time can be related to the material Deborah (De) number: the specimen reaches steady deformation behavior when the observation (measurement) time is larger than the invariant time.

The test data analyzed in this work were divided into two classes: pseudo-static (creep) and dynamic. For creep under constant applied stress, the method [27,56,57] predicts a log-time dependence of the strain with *E*^0^_c_ as the averaged invariant creep modulus, *τ*^0^_c_—invariant characteristic creep time, *α*_c_—material creep memory parameter. For dynamic loading case, the method predicts a sub-dimensional dependence of the frequency for the strain amplitude [27,46,57]. When the input stress is harmonic, the strain depends on observation time t, frequency ω and constant stress amplitude *σ*_dyn_, with *E*^0c^_ω_ as the averaged invariant dynamic modulus (i.e., not dependent on time and frequency), *τ*^0^_ω_—invariant characteristic dynamic time, *α*_ω_—dynamic material memory parameter. This Equation (8) comprises the time-convolution of the specimen loading history without a general need of complex algebra, assumptions of a material model (Maxwell, Burger, standard linear solid, Prony series, Mooney–Rivlin, Ogden, hyperelastic, neo-Hookean, etc.) or use of local differentiation [20,23,58,59].

The values of *E^0^*, *τ^0^* and *α* are time-invariant, in a sense that they do not depend on the time of experiment. Equation (8) can be numerically, explicitly computed without the need for assumptions of linearity of *E^0^*, *τ^0^* and *α*, whether they are for a creep (static) or dynamic case. According to [20,27], this allows one to overcome common restrictions for the linearity of tissue properties in many models [19], namely a scaling property (homogeneity) and a superposition property (additivity) (addition and multiplication are two linear operations which are applicable to complex numbers-in general, to the commutative rings [58,60]). This might not generally hold for (linear) Fourier transformation, commonly used in linear viscoelasticity, as most tissues and biomaterials properties functions are frequency- or strain-rate-dependent [61,62,63]. For example, if the loss tangent, commonly expressed as ratio of imaginary to real moduli in viscoelasticity, is not constant, a rigorous application of complex math transformation cannot be made correctly, leading to wrong predictions, artefacts or just improper conclusions [62]. Authors [63] have underlined that the presentation of oscillatory stress or strain response in a real Fourier series is not at all equivalent to complex Fourier transformation, especially when only the first harmonic real and imaginary moduli are calculated as this itself imposes linearity transformation requirements (in the real world, there are no complex physical quantities: it is not possible to have a mass like 3 + 2*i* kg or time like 10 − 3*i* seconds—so no complex moduli or viscosity could exist in reality, being just mathematical abstractions).

For the time-convolution Equation (6), such linearity conditions assumption is not required due to idempotent processing [60]: the values of these memory parameters are always positive (because of causality principle: no response is generated before the stimulus has been applied). When they are in the range 0 < *α* < 1, they represent the fading memory. The smaller the value, the greater is the effect of short-time memory (= immediate reaction to the stimulus). Zero value means that the material has only a short-time memory, i.e., ideally elastic behavior, whereas *α* = 1 means ideally viscous behavior [27,46,56,57,59]. For the cases 1 < *α* < 2, the material only “feels” a long-term memory and the immediate elastic reaction on the stimulus is damped: the material mechanical behavior could turn into inertial damped oscillations [27,57].

It is noteworthy to emphasize that invariant values of *E^0^, τ^0^* and *α* describe the whole system behavior (tissue components, its fluids, possibly with blood content, etc.) interacting with the applied mechanical stimuli, and not the single materials proprieties values. This is different from the traditional way of mechanistic description of every tissue component (like collagen fibrils, extracellular matrix, etc.) separately, followed by assembling these descriptions into some set of approximate equations.

### 3.3. Statistical Method and Data Visualization

The data assessment procedure for soft tissues after digitizing literature data was realized in this work as follows. First, the presence of leverage points was detected with hat matrix diagonal components, which were not falling under Stephen’s rule (these points were removed). An influencer (outliers) points analysis was made by calculating Cook’s distances, and those data points exceeding unity value (if any) were also removed. The consistency of obtained coefficients with quantum regression method (Seqvera Ltd., Helsinki, Finland) was independently checked by the application of the Theil–Shen estimator and the goodness of fit significance—by the Nelson-modified Anderson–Darling test [64] (data not shown: the original algorithm in [64] assumes all positive input numbers and fails if a negative normalized value appears in the data. This feature has been improved in the present analysis). The heteroscedasticity of residuals was estimated with RUNS test and possible residuals’ autocorrelation—with Durbin–Watson parameter. 

All the data passed these criteria were used in Equation (8) to obtain biomechanical invariant values [24], which were considered to be BLUE (BEST linear unbiased estimators). This calculation method does not impose linearity, scaling and superposition requirements for soft tissues [19,36,37]. A visualization of moduli rotation in 3D by Equations (4) was made with MathCAD 14 software (PTC Inc., Needham, MA, USA). This procedure comprises the calculation of the bone stiffness matrices as Equations (1)–(3), stepwise “blue” rotation of the tensors by 0~90° anticlockwise around *Z*-axis, followed by “red” rotation by 0~90° around X’ axes (Figure 1), recalculation of the rotated stiffness for each rotation step with Equations (4), inversion of the stiffness tensor Equation (1) to obtain the compliance matrix, the calculation of respective Poisson ratio components (3b) and the 3D plotting of the calculated values.

### 3.4. Clinical Case Simulation

**Figure 2:**
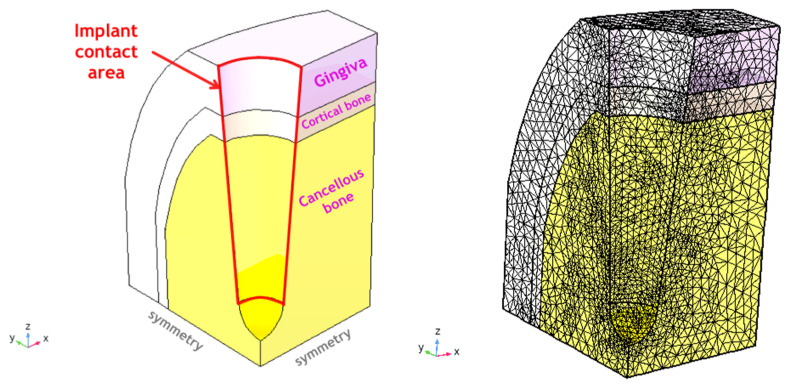
Simulation domain (the ¼ symmetry part of the full domain; left) and the mesh used (right) with COMSOL 5.4 (Comsol Inc., Burlington, MA, USA). The mesh consists of 191,615 elements with a higher density closer to implant surface and has 804,187 degrees of freedom to be solved for. The computational domain size is 5 × 6 × 10 mm^3^ (X × Y × Z) with boundary conditions shown above.

Based on the estimated tissues properties, a simulation of the clinical case has been made with the following conditions. We assumed that a patient CT-scan has been performed, pre-assessed by the doctor and the primary tissue data (geometry, density, size, location) have been extracted. A mandibular implant is placed at the molar position into normal quality bone (along unrotated Z-axis, Figure 1). Cortical bone thickness was taken as 1 mm and its density as 1.8 g/cm^3^; medullar (cancellous) bone thickness as 6.5 mm with its density 600 HU (the apparent density of 0.607 g/cm^3^ as by Equation (5)). Gingival thickness is taken as 2 mm average and its properties are approximated with BEST method, as shown in Equation (8). The implant micromotion amplitude in Z-direction was set to 50 μm at 1 Hz, imposed on the axially symmetric surface (Figure 2) leading to traction along the XZ- and YZ-planes (Figure 1). Due to the symmetry, the same traction displacement will also act in rotated planes X’Z and Y’Z (Figure 1), but not in X’Y”, X’Z” or Y”Z” planes, which require a rotation conversion by Equations (4) first.

All tissues (cancellous bone, cortical bone, gingiva) were assumed to face the same micromotion as a boundary condition at the interface (= there is no assumption on the adhesion quality of the screw and the abutment, the traction is supposed just to be transferred to the contact surface). All materials (tissues) were set to “linear elastic material” in the COMSOL software, but with the customized properties described for every matrix element as by Equations (5)–(8) introducing the non-linearity and history-dependent viscoelasticity. As initial data for bone in Table 1 did not have viscoelastic components, a loss factor of 0.03 was adopted for 1 Hz based on general knowledge [9,16,21]. For gingival tissue, non-linear viscoelastic contribution was embedded in the stiffness matrix with Equation (8). The thread and shape of the screw are not included in the calculations for this demonstration (as they are implant-type specific). The base XY plane is assigned with roller conditions (no vertical Z-displacements) and symmetry planes imposed at XY and YZ planes—in this way, ¼ of the whole structure is simulated. All other surfaces are set to free displacements (Figure 2).

## 4. Results

### 4.1. Stiffness Estimation and Visualization for an Arbitrary Direction

**Figure 3:**
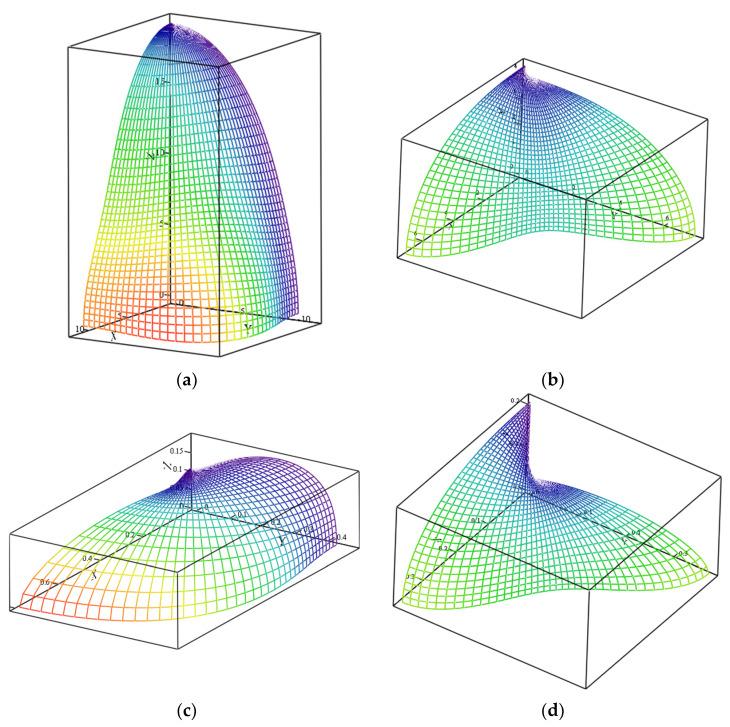
Vizualized elastic (E_11_; **a**,**c**) and shear (G_12_; **b**,**d**) moduli (GPa) for cortical bone with density of 1.8 g/cm^3^ (**a**,**b**) and 600 HU density cancellous bone (**c**,**d**).

The estimation of the stiffness (2) and compliance (3a) matrices was calculated for fixed bone densities, as shown in Section 3.2 with a stepwise (40 steps) rotation by 90°, first around the Z-axis and then around the X-axis (Figure 1). For every step, the tensor components (2) were recalculated and then converted into components of elastic moduli (here viscous contribution was introduced with Equation (8) for every matrix component). Examples of moduli for XX (E_11_) and shear XY (G_12_) are shown in Figure 3 in phase space. The coordinate system reflects the unrotated (initial) state, and the point of view—respective rotation.

For example, for Figure 3a (E_11_), the view vector is directed from the coordinates origin towards the reader, corresponding to the rotations by yaw Ψ~30° and pitch Θ~15° (Figure 1). The resulting stiffness matrix **C** and respective Poisson ratios matrix for this case for cortical bone is shown matrixes (9a, 9b), where C values are in GPa and Poisson ratios matrix for cortical bone, as seen from the point of view of Figure 3a (orthogonally to the picture: from the reader towards coordinates origin). It is seen how stiffness anisotropy affects moduli depending on which direction it might be seen. All components of the **C** matrix (9a) are now non-zero as they are off principal axes. Despite being the dynamic simulation (1 Hz), the stiffness matrix components and Poisson ratios (9b) are all real, as due to method by Equation (8), no complex math via Fourier transform was needed.

The literature data for gingival tissues were processed with Equation (8) and the gingival/mucosal invariant data have been extracted (Table 5). Analyzed creep values were consistent in the invariant time scale (13~17 s) but differ in creep modulus, possibly due to about 200 times difference in applied stress in those experiments. In dynamic experiments, the data showed the opposite trend: the invariant moduli values are compatible, but invariant times have much higher difference, Table 5. This can be explained by dissimilar experimental conditions and that in [46] gingiva was not separated but kept attached to the underlying bone and submitted to a wide strains range (up to 0.4). These last data were used in in silico simulation for the gingival part of the model, as they are the most coherent for the clinical case considered.

(9a) $$∥C{∥}_{cort.\;rotated}\;=\;∥\begin{array}{cccccc} 17.715 & 4.826 & 6.284 & 0.218 & -0.864 & -1.129\\ 3.968 & 16.908 & 4.918 & -0.418 & 0.274 & 1.559\\ 14.67 & 14.076 & 25.365 & 0.082 & 0.652 & 0.012\\ 0.171 & -0.466 & 0.054 & 4.445 & -0.216 & -0.141\\ 1.557 & 2.67 & 3.073 & -0.222 & 4.656 & 0.28\\ -0.954 & 1.739 & 0.114 & -0.141 & 0.259 & 4.933 \end{array}∥$$

(9b) $$∥v{∥}_{cort.\;rotated}\;=\;∥\begin{array}{cccccc} 1 & 0.139 & 0.317 & 0.013 & -0.071 & -0.089\\ 0.107 & 1 & 0.212 & -0.03 & 0.011 & 0.116\\ 0.52 & 0.47 & 1 & 0.013 & 0.061 & -0.013\\ 0.033 & -0.095 & 0.043 & 1 & -0.044 & -0.014\\ -0.054 & 0.191 & 0.435 & -0.043 & 1 & 0.031\\ -0.239 & 0.356 & -0.012 & -0.014 & 0.032 & 1 \end{array}∥$$

### 4.2. In Silico Simulation

**Figure 4:**
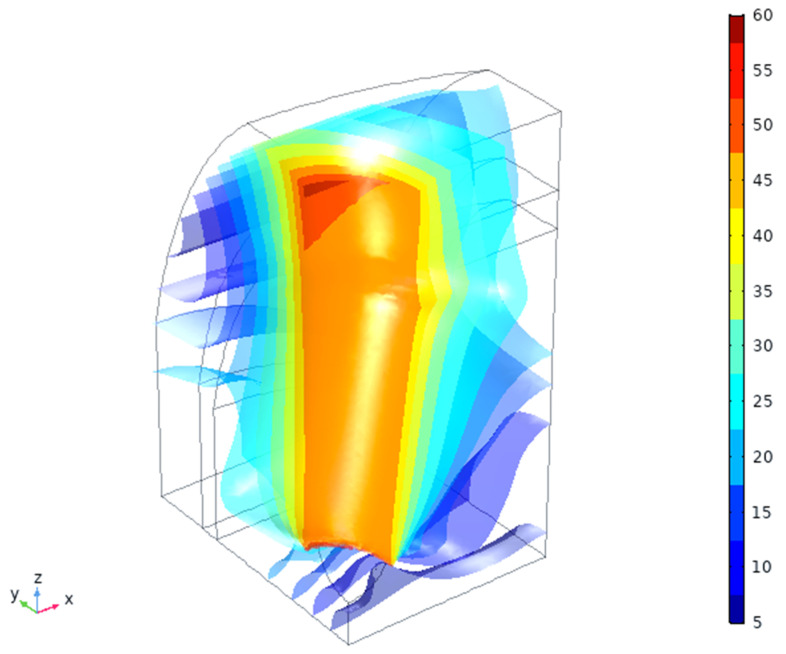
Total displacement magnitude (μm) of the tissues vs. applied 50 μm amplitude.

**Figure 5:**
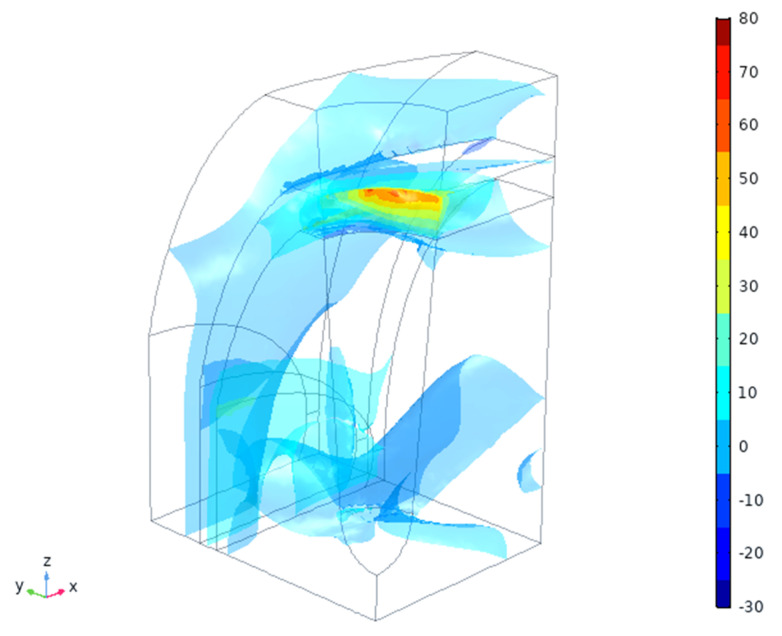
Expected hydrostatic pressures in the tissues (MPa).

The total displacement amplitude of the tissues (μm) is shown in Figure 4. As expected, there are non-uniform, anisotropic displacements in all three tissue types, being the highest near to the implant traction surface—contact area (Figure 2, right). The maximal positive pressure appears near this interface and is the highest in the cortical bone in the X-direction (mesio-distal), Figure 5. This zone, however, is rather small (~100 μm depth) and changes into negative pressure zone deeper in the tissues. Neutral zone stays in gingival tissue and in the deeper cancellous bone, which might be considered positive from the point of view of having lower PPT and IFP values (Table 3). A detailed analysis of these threshold zones is possible, but it likely makes more sense for an exact implant shape and thread geometry.

**Figure 6:**
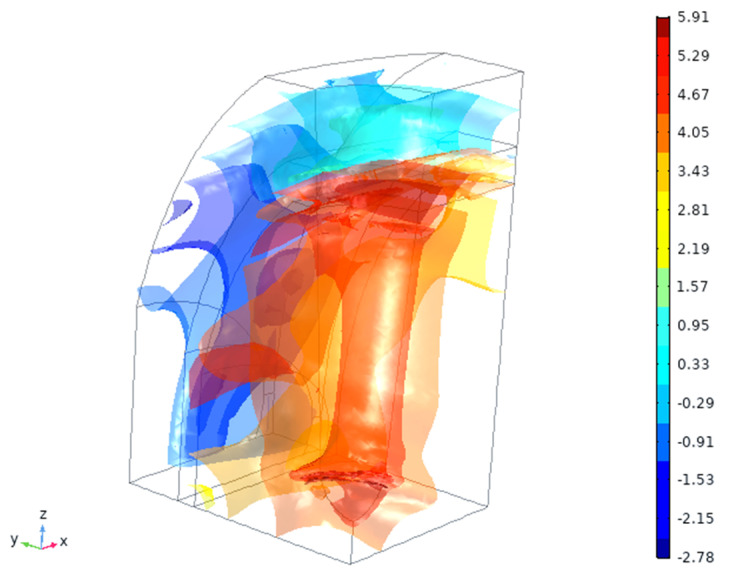
Calculated strain energy density (log [J/mm^3^]) in the tissues.

Bone is the tissue which bears most of the load, as seen from the strain energy density by 4–6 orders of magnitude (Figure 6). There is also a noticeable gradient of strain energy density (by ~1000 times) inside the gingival tissue. The values of the strain energy density magnitude in gingiva are, however, small (as there are very little deformation, Figure 4), so they are unlikely to cause any intermittent effects. On the other hand, long-term effects might be noticeable if the strain redistribution will proceed with time. 

Simulation can also show different specific stress and strain components and other variables, as well as their time variation within the loading cycle (~1 Hz) or in a longer scale (creep) for detailed analysis. It might be interesting for clinical cases to identify possible critical areas and risk points, especially for personalized shapes of the implant—for example, a small, 0.2~0.5 mm change in thread or diameter in some areas might have a significant impact on clinical success.

## 5. Discussion

In this work, a simulation approach has been presented comprising a combined application of non-linear anisotropic tissue descriptions with semi-analytical and numerical computation. Here, the last one formally deploys an embedded “linear elastic material” description (a standard FEA feature), having however non-linear, invariant components of the stiffness tensor. Such methods are usually referred to as “reduced order models”, capable of a very quick operation for adequate description of complex systems involving multi-phase, multi-materials combination in real-time dynamics [65,66]. This has a clear advantage over sophisticated non-linear methods—for example, in [67] it was assessed that for an incompressible second-order reduced polynomial model with a second-order quasi-linear viscoelastic extension in Prony series (with four unknowns), the analysis of the test data took about 24–40 h of computational time on a standard desktop computer. For ten or fifteen unknowns and a higher mesh density (more degrees of freedom), such an approach is unfeasible even with sufficient computational power.

The implant design and selection are highly personalized and present an important part of the whole implantation procedure. Therefore, for practical reasons, it is desirable that collected data and patient conditions could be used for such selection in a fast and safe way. It is less likely that a doctor or dental lab would engage themselves in sophisticated computations, taking time and generating a substantial amount of data needed to be analyzed, filtered and converted into decision making.

Here, numerical data compression has been achieved with the time convolution method and complemented by algorithm allowing visualization of the tensor components in 3D-rotated coordinates. Whereas the stiffness component visualization is not obligatory for the implant design and selection, it helps us to see the directions where the tissues stiffness is e.g., the weakest (zones of increased risks). Such visualization assists the user in the understanding of the tissues’ anisotropy and its variation vs. view angle or direction of load application in 3D. The latter might be considered, for example, as a direction in which the force is applied to give an immediate response how this force would be transformed into respective strain components and pressure (important for PPT, IFP and residual ridge resorption; Table 3).

The most significant findings of the present work are:

- Transitions from the single “elastic modulus” values towards true stiffness matrix components, which are real material functions of density, strain, time, frequency, etc. give a more precise and comprehensive description of the tissues with patient-specific data (such as bone density in loco).
- Visualized rotation and generated 3D plots can be of an assistance of identification of the weaker and stronger directions in the X-Y-Z coordinates that may help the digital planning of surgery (for example, where the lowest stiffness part might coincide with thinner or lighter bone in that direction).
- For soft tissues (gingiva, mucosa), invariant values (modulus *E*, characteristic time *τ* and material memory *α*) have shown being extracted from experimental data without the assumption of a material model. The same can be made for bone, if viscoelastic effects need to be considered without the need of complex numbers processing.
- In the case shown, the data used were obtained closer to the physiological range, allowed to mimic the behavior without needs to break down the values into specific tissue components. These invariant values comprise time-convoluted data and are better predictors for materials performance comparison than traditional stiffness (stress/strain ratios).
- Non-linear viscostiffness data can be placed into software packages for a detailed simulation, and there, the directional rotations are performed automatically. Here, the detailed simulation of the implant screw and abutment is not always necessary—the imprint surface transferring the load is of the greatest interest as a boundary condition. By changing the patient-specific parameters, a user can simulate a number of relevant scenarios in a short time to see whether a specific implant in that location would potentially have risky zones. This can simplify calculations for more realistic clinical cases with 3D implant placement planning and outcome estimation.

The algorithm for the deployment of this method in practice might be as follows:

- Examining the patient and determination of the bone/tissues status (quality) and parameters for the implantation site.
- Selection of the implant type (dictated by the general considerations and safety).
- Entering the patient’s parameters and implant geometry into the 3D model.
- Setting up “normal” (a nearly perfect osseointegration) or “worst case” (e.g., absence of cohesion between the screw or the abutment) scenarios and performing numerical calculations.
- Post-processing the results, leading to the identification of potential risk zones and parameters.
- In the case of need, adjustment or changing the implant type, shape, thread geometry, etc. and repeating the computation for the optimal outcome.

The limitations of the recent study are linked to likely over-generalized case of the tissues’ properties: for the bone, the data scatter is lower, but soft tissues have much more variations. Authors might recommend that all the relevant tissues biomechanical properties should be tested in the conditions as possibly close to the real situation, and not in artificially created ones (even if such conditions were considered as a standard). In the present analysis, screw thread and details of the implant surface were not considered, as they require more detailed meshing and more computations. For realistic patient cases, the use of ¼ of the full tissue can be too simplified as the mandible (as well as maxilla and other sites) are not fully symmetric at XZ and YZ planes (Figure 1).

For the analysis of literature data, there was little possibility to validate those test conditions and the data reliability coming from very different sources. Authors [63] have warned that in many published datasets, issues related to inertia effects (high frequencies) or instrument torque limits (low frequencies) are usually not sufficiently documented. Hence, even if instrument inertia is eliminated, the sample itself will always have finite inertia, which can produce artefacts from momentum diffusion, viscoelastic waves, and secondary flows, all of which can violate the assumption of homogeneous and linear deformation [63]. The data in Table 1 were elastic only, and in Table 2, have a limited number of values for viscoelastic parameters. In this work, these data were assumed as sufficiently credible, even when no additional supporting information had been provided. This limits the outcomes in Section 4 to the level of the initial data quality, but definitely can be improved with better initial values.

## 6. Conclusions

1. Correct biomechanical description of oral tissues has to be based on realistic stiffness matrix components, being functions of density, strain, time, frequency etc.—and not on a single value of “elastic modulus”.
2. Consistent invariant values (modulus, characteristic time, material memory) can be extracted from experimental data without the assumption of a material model, when these experiments are carried out closer to physiological range (mimicking the behavior of the tissue, implant or tissue-implant assembly). Such invariant values are better predictors for the performance comparison than traditional stiffness (stress/strain ratios).
3. Known non-linear viscostiffness data can be placed into commercial software for a detailed simulation with automatic directional rotations to obtain the distribution of stresses, strains, strain energy density, pressure etc. for better decision-making.
4. The approach based on a combined application of non-linear anisotropic tissue descriptions, as a reduced order “model-free” method (i.e., not linked to a priori chosen model), is capable of a very quick operation (“data compression”) for an adequate computation of multi-tissue behavior in the contact with the dental implant in real-time dynamics. The approach can be expended to real screw and abutment geometry for securing better outcomes in planning dental implant operations.

## Figures and Tables

**Table 1:** Some reported data for elastic modulus of cortical and cancellous bones.

Method [Reference]	Tissue	Results *	Comments
Various data [28]	Cortical bone	14.0	Data adopted for finite element analysis (FEA), bone density 1.3–2.0 g/cm^3^
		13.0	
		13.7	
Various data [31]	Cortical bone	11.4~14.1	Buckling
		5.72	Bending
		13.0~14.8	Ultrasound (human)
		10.9	Ultrasound (bovine)
		10.4	Tensile
		15.0~19.4	Nanoindentation
		17.4~17.5	Acoustic microscopy
[29]	Cortical bone	12.6~19.4	Orthotropic formulations
		Shear 4.85~5.7	
[32]	Cortical bone	13.98	Poisson ratio 0.30
Calculated and analyzed in [33]	Cortical bone	13.7~15	At density 1.8 g/cm^3^
		15~20	Infero-superior direction (Z)
		8~10	Bucco-lingual direction (Y)
		9.8~10	Mesio-distal direction (X)
Assigned by bone density [30]	Bone type I	9.5	Mandible (anterior)
	Bone Type II	5.5	Mandible & maxilla (anterior)
	Bone Type III	1.6	Mandible (posterior), maxilla (anterior)
	Bone Type IV	0.69	Maxilla (posterior)
Various data [31]	Trabecular bone	0.76	Uniaxial tension
Various data [28]	Cancellous bone	0.49	Data adopted for finite element analysis (FEA), density 1.0 g/cm^3^
		1.37	
		0.50	
		0.69	
[32]	Medullar bone	0.259	For low density (150 HU)
		3.507	For high density (850 HU)
Adopted in [29]	Cancellous bone	0.210~1.148	Orthotropic formulations
		Shear 0.068~0.434	
Calculated and analyzed in [33]	Cancellous bone	0.20~0.25	At density 1.0 g/cm^3^
		0.30~0.33	Infero-superior direction (Z)
		1.0~1.7	Bucco-lingual direction (Y)
		3~4	Mesio-distal direction (X)

* Values are in GPa.

**Table 2:** Reported experimental data for soft oral tissues. Tissue origin: H = human, P = porcine; B. = buccal, L. = lingual. Modulus values in MPa and their standard deviations, unless shown otherwise.

Method [Reference]	Tissue	Results	Comments
Tension at 22 °C with stress/strain analysis (20 mm/min) [39]	Gingiva (H)	37.36 ± 17.36	No data about calculation. Preconditioning 0.5–2.0 N with 20 cycles. Specimens from Thiel-embalmed cadavers
	B. mucosa (H)	18.13 ± 4.51	
	Hard palate (H)	8.33 ± 5.78	
Tension with preload 3 mN (5 mm/min) [40]	L. attached gingiva (P)	18.83 ± 5.98	Preconditioning with 5 cycles before load to failure
	B. attached gingiva (P)	19.75 ± 6.20	
	L. alveolar mucosa (P)	4.79 ± 2.54	
	B. alveolar mucosa (P)	5.74 ± 1.15	
	B. mucosa (P)	2.48 ± 0.37	
Compression at 0.1–1 Hz, 10–15% strain [40]	B. attached gingiva (P)	‘Instant’ 7.81 ± 1.11	Preconditioning: 25 cycles + 5 min relaxation. Computed at the peak stress (1st and last cycle)
		‘Steady’ 0.86 ± 0.09	
Compressive creep 0.36 kPa, 37 °C in 5 min [38]	Hydrated mucosa (P)	2.72	Creep and relaxation were not analyzed in detail
	Dehydrated mucosa (P)	E’ 2.0~4.5	Data for E’ at 1 Hz, 37 °C
		E’ 0.2~0.35	-
Various data [17]	Mucosa (H)	Creep: 0.04~2.35	Data from different sources
		Initial: 0.083 ± 0.020	not all Initial values is creep for 1st Prony series
		Linear: 0.1~680	Poisson ratios: 0.30~0.49
		Others: 1~10, 19.6	-
		Standard linear solid: 1.1, 1.2	For two spring elements
In vivo MRE [37]	Soft palate (H)	G’ 2.53 ± 0.31	Shear moduli from the MRE displacements
		G” 0.90 ± 0.22	
Various data [26]	Mucosa (H)	0.91~5.93	Measured by ultrasound
		0.37~5.80	Mechanical measurements
		0.41~2.67	-
		0.66~4.36	-
		2.75~5.03	-
		0.37~0.59	Kelvin–Voigt model
		1.41	Maxwell model
Various data [19]; mechanical tests	PDL (P)	0.070~1750	Different measurements
		5.5 ± 2.1	At strain rate 0.002 L/s
		12.5 ± 4.2	At strain rate 0.04 L/s
		19.0 ± 6.3	At strain rate 1.2 L/s

**Table 3:** Parameters of clinical relevance related to biomechanics (in kPa).

Parameter (Purpose)	Preferred Values	Maximal (Short-Term) Values	Refs.
PPT (minimize pain)	<100	<400	[17]
IFP (minimize anoxia/ischaemia)	<4	<13	[17,47,48]
Pressure (minimize residual ridge resorption)	<7	<20	[17,46]

**Table 4:** Reported data for unrotated stiffness tensor components (GPa) and Poisson ratios for cortical bones [29,31,33]. Data in *italics* in shaded cells are calculated in this work.

Type	*C*_11_	*C*_22_	*C*_33_	*C*_44_	*C*_55_	*C*_66_	*C*_12_	*C*_13_	*C*_23_	*ν*_12_	*ν*_23_	*ν*_31_	*ν*_21_	*ν*_32_	***ν*** **_13_**
Bovine femur	14.1	18.4	25.0	7.00	6.30	5.28	6.34	4.84	6.94	*0.38*	*0.206*	*0.306*	*0.303*	*0.109*	*0.204*
Human tibia	11.6	14.4	22.5	4.91	3.56	2.41	7.95	6.10	6.92	*0.61*	*0.316*	*0.306*	*0.495*	*0.119*	*0.142*
Human femur	20.0	21.7	30.0	6.56	5.85	4.74	10.9	11.5	11.5	*0.021*	*0.474*	*0.426*	*0.019*	*0.376*	*0.375*
Bovine femur Haversian	21.2	21.0	29.0	6.30	6.30	5.40	11.7	12.7	11.1	*0.437*	*0.434*	*0.281*	*0.408*	*0.282*	*0.191*
Bovine femur plexiform	22.4	25.0	35.0	8.20	7.10	6.10	14.0	15.8	13.6	*0.515*	*0.562*	*0.229*	*0.399*	*0.297*	*0.156*
[33]	*15.73*	*18.59*	*26.91*	4.63	4.31	3.81	*8.16*	*9.68*	*9.73*	0.381	0.445	0.328	0.309	0.249	0.224
[29]	*17.38*	*17.38*	*27.02*	5.7	5.7	4.85	*7.69*	*6.34*	*6.34*	0.30	0.253	0.253	0.30	0.39	0.39

**Table 5:** Calculated invariant values from published experimental data for soft oral tissues. Deviations (where shown) are based on published data points scatter.

References	Measurements	Results	Comments
[34,35]	Creep at 83 kPa	E^0^_c_ = 73 ± 21 kPa τ^0^_c_ = 17 ± 3 s	Only one creep stress
[38]	Creep at 0.36 kPa in compression, 37 °C	E^0^_c_ = 262.8 kPa τ^0^_c_ = 13.2 s	Only one stress used; limited test time (5 min)
[40]	Dynamic compression at 1 Hz and 0.1 strain	E^0^_ω_ = 272.7 kPa τ^0^_ω_ = ~0.02 ms?	Only 0.1 strain data shown; large data scatter
[46]	Special shear simulating abutment load at 1 Hz and 37 °C	E^0^_ω_ = 117 ± 8 kPa τ^0^_ω_ = 23 s	Gingiva attached to the bone support; up to 50 μm displacement

## Data Availability

Data sharing is not applicable to this article.
